# An N-terminally truncated envelope protein encoded by a human endogenous retrovirus W locus on chromosome Xq22.3

**DOI:** 10.1186/1742-4690-7-69

**Published:** 2010-08-24

**Authors:** Christina Roebke, Silke Wahl, Georg Laufer, Christine Stadelmann, Marlies Sauter, Nikolaus Mueller-Lantzsch, Jens Mayer, Klemens Ruprecht

**Affiliations:** 1 Institut für Virologie, Gebäude 47, Universitätsklinikum des Saarlandes, 66421 Homburg, Germany; 2 Institut für Neuropathologie, Georg-August-Universität Göttingen, Robert Koch Strasse 40, 37075 Göttingen, Germany; 3 Institut für Humangenetik, Gebäude 60, Universitätsklinikum des Saarlandes, 66421 Homburg, Germany; 4 Klinik für Neurologie, Charité Universitätsmedizin Berlin, Campus Charité Mitte, Charitéplatz 1, 10117 Berlin, Germany

## Abstract

**Background:**

We previously showed that the envelope (*env*) sequence of a human endogenous retrovirus (HERV)-W locus on chromosome Xq22.3 is transcribed in human peripheral blood mononuclear cells. The *env *open reading frame (ORF) of this locus is interrupted by a premature stop at codon 39, but otherwise harbors a long ORF for an N-terminally truncated 475 amino acid Env protein, starting at an in-frame ATG at codon 68. We set out to characterize the protein encoded by that ORF.

**Results:**

Transient expression of the 475 amino acid Xq22.3 HERV-W *env *ORF produced an N-terminally truncated HERV-W Env protein, as detected by the monoclonal anti-HERV-W Env antibodies 6A2B2 and 13H5A5. Remarkably, reversion of the stop at codon 39 in Xq22.3 HERV-W *env *reconstituted a full-length HERV-W Xq22.3 Env protein. Similar to the full-length HERV-W Env protein Syncytin-1, reconstituted full-length Xq22.3 HERV-W Env is glycosylated, forms oligomers, and is expressed at the cell surface. In contrast, Xq22.3 HERV-W Env is unglycosylated, does not form oligomers, and is located intracellularly, probably due to lack of a signal peptide. Finally, we reconfirm by immunohistochemistry that monoclonal antibody 6A2B2 detects an antigen expressed in placenta and multiple sclerosis brain lesions.

**Conclusions:**

A partially defective HERV-W *env *gene located on chromosome Xq22.3, which we propose to designate ERVWE2, has retained coding capacity and can produce *ex vivo *an N-terminally truncated Env protein, named N-Trenv. Detection of an antigen by 6A2B2 in placenta and multiple sclerosis lesions opens the possibility that N-Trenv could be expressed *in vivo*. More generally, our findings are compatible with the idea that defective HERV elements may be capable of producing incomplete HERV proteins that, speculatively, may exert functions in human physiology or pathology.

## Background

Multiple sclerosis (MS) is a chronic inflammatory demyelinating disease of the central nervous system affecting primarily young adults. While its precise aetiology is unknown, MS is thought to be a multifactorial disorder resulting from the interaction of environmental and genetic factors [1]. A multiple sclerosis-associated retrovirus (MSRV) has previously been suggested by cDNA clones that were generated from particle-associated RNA from plasma or supernatants of cultured cells from patients with MS [2-4]. Subsequent investigations revealed MSRV-related sequences in the human genome, the human endogenous retrovirus family type W (HERV-W) [5].

HERVs are considered remnants of ancestral germ line infections by once active retroviruses and contribute approximately 8% of the human genome (for review see [6,7]). Like their exogenous counterparts, HERVs typically consist of an internal region containing *gag*, *pro*, *pol*, and *env *genes, flanked by two long terminal repeats (LTR). The number and phylogenetic relationships among HERV-W sequences in the human genome have been addressed before [8,9]. HERV-W is a multicopy endogenous retroviral family comprising approximately 650 elements. About 280 of those elements contain internal sequences [8]. Individual HERV-W loci are defective due to the acquisition of stop-codons, truncations, and deletions. In addition, many HERV-W elements actually represent processed pseudogenes resulting from retrotransposition by long interspersed element (LINE) machinery [8,9]. De Parseval *et al*. identified 13 HERV-W *env *elements in the human genome with full-length *env *genes [10]. Among these, only one HERV-W *env *locus on chromosome 7q21.2, named ERVWE1, has retained an uninterrupted open reading frame (ORF) for a functional envelope (Env) protein, termed Syncytin-1, which is likely involved in placental morphogenesis [10,11].

A number of previous reports have suggested a possible role of Syncytin-1 and/or MSRV Env protein in the pathogenesis of MS [4,12-19]. MSRV/HERV-W *env *RNA is more abundant in autopsied brain tissue from patients with MS than from controls [12,16,17,19]. A monoclonal anti-HERV-W Env antibody (mAb 6A2B2) detects an antigen expressed in actively demyelinating brain lesions from patients with MS [12,16,18]. Importantly, expression of Syncytin-1 in astrocytes induces release of mediators that are cytotoxic to oligodendrocytes (the cells responsible for myelination) *in vitro*, and the expression of Syncytin-1 in murine models causes oligodendrocyte loss and demyelination *in vivo *[16,18]. On the other hand, MSRV Env protein (AAK18189.1) has been suggested to have superantigen-like properties [4], and the surface (SU) domain of MSRV Env, which is 87% identical to Syncytin-1, was reported to have proinflammatory effects via activation of CD14 and toll-like receptor 4 [15].

Despite the potential involvement of Syncytin-1 and MSRV Env in MS, the precise origin of MSRV *env *sequences and their relation to endogenous HERV-W *env *loci has not been clear [19-23]. We recently proposed that formerly reported MSRV *env *sequences may be explained as being derived from transcripts of various genomic HERV-W *env *loci or from recombinations among those transcripts [24]. By analogy to data obtained from a study of transcribed HERV-W *env *loci in human peripheral blood mononuclear cells (PBMC), and from a study of transcribed HERV-K(HML-2) loci, it seems possible that those recombinations occurred *in vitro *because of template switches of reverse transcriptase during cDNA generation and/or via PCR-mediated recombinations [24,25].

In particular, our analyses showed that the SU region and the 5' part of the transmembrane (TM) region of the reported MSRV *env *sequence AF331500 are highly similar to a HERV-W *env *locus on human chromosome Xq22.3, while the 3' part of the TM region of AF331500 is highly similar to a HERV-W element on chromosome 5p12. Another published MSRV *env *sequence (AF127228) was found to be almost identical with the HERV-W locus on chromosome Xq22.3 as well [24]. The Xq22.3 HERV-W *env *locus is quite remarkable as it harbors an almost complete ORF for a full-length HERV-W Env protein, only interrupted by a single premature stop at codon 39. The longest possible ORF of HERV-W Xq22.3 *env*, starting at an in-frame ATG at codon 68, could produce an N-terminally truncated HERV-W Env protein of 475 amino acids. Others and we previously showed that Xq22.3 HERV-W *env *is transcribed in human PBMC [24,26,27]. Similar to other transcribed HERV-W elements, the Xq22.3 locus lacks a 5'LTR promotor, suggesting that another upstream promotor drives its transcription [26,27]. While that promotor remains to be identified, Xq22.3 HERV-W *env *transcripts indicate that the locus fulfills one essential prerequisite for protein production.

Intriguingly, it turned out that anti-HERV-W Env mAb 6A2B2 (detecting an antigen in MS brain lesions, see above) was raised against a 379 amino acid sequence encoded by MSRV *env *clone AF127228, which, except for two C-terminal amino acid exchanges, is identical to the Xq22.3 HERV-W Env amino acid sequence [11,24]. Although 6A2B2 may crossreact with Syncytin-1 [11,28,29], these findings open up the possibility that the protein detected by 6A2B2 in MS lesions may in fact be derived from Xq22.3 HERV-W *env*. Nonetheless, it was unknown whether a protein encoded by Xq22.3 HERV-W *env *can be expressed in human cells.

We herein show that Xq22.3 HERV-W *env *is capable of producing an N-terminally truncated HERV-W Env protein *ex vivo*. Reversion of the stop codon at position 39 in Xq22.3 HERV-W *env *results in the expression of a reconstituted full-length HERV-W Env protein. We characterize properties of truncated and reconstituted Xq22.3 HERV-W Env in comparison to Syncytin-1 and MSRV Env. We also confirm that mAb 6A2B2 detects an antigen expressed in placenta and MS brain lesions. Our data support the idea that not only HERVs with ORFs for complete retroviral proteins but also defective HERV elements may be capable of producing pieces of HERV proteins, which, speculatively, may exert functions in human physiology or pathology.

## Results

### Expression of an N-terminally truncated Env protein from HERV-W Xq22.3

**Figure 1:**
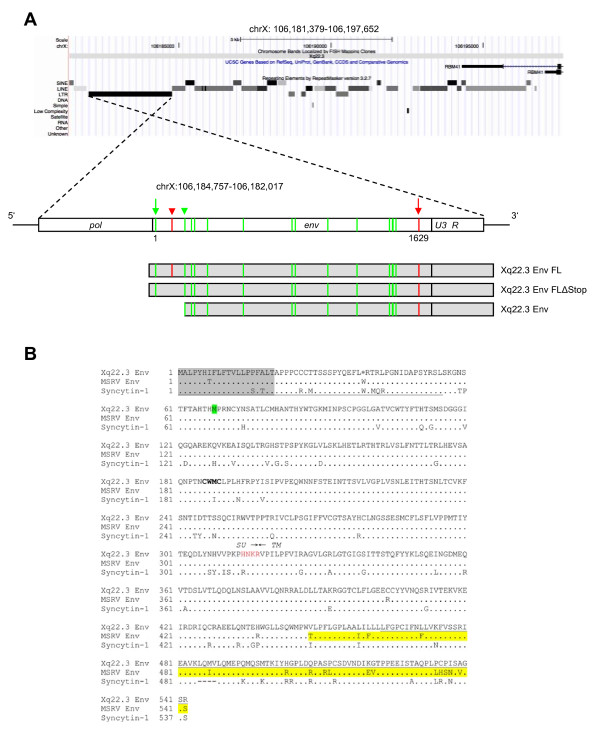
**Structure of the Xq22.3 HERV-W locus**. (A) Organization of the HERV-W locus on human chromosome Xq22.3. Shown on the top is a screenshot from the UCSC Human Genome Browser [39] depicting the chromosome × region of interest and the flanking *RBM41 *gene. Note that the Xq22.3 HERV-W locus is located in antisense orientation on the chromosome. Start and stop codons within the *env *region are indicated by green and red vertical lines. The start (nt 1) and end of the 1629 nt long *env *ORF are marked by green and red arrows. The stop codon at position 39 and the start codon at codon 68 are marked by a red and a green arrowhead. Portions of the Xq22.3 HERV-W locus that were inserted into the phCMV expression vector for subsequent *in vitro *studies are depicted below. (B) Amino acid sequence alignment of Xq22.3 Env, MSRV Env (AAK18189.1), and Syncytin-1 (NP_055405.3). Signal peptides (SignalP 3.0, http://www.cbs.dtu.dk/services/SignalP) are shaded in gray. The stop codon at position 39 of Xq22.3 HERV-W Env is indicated by an asterisk (*) and the start codon at position 68 is highlighted in green. The consensus C-X-X-C motif is shown in boldface. The boundary between SU and TM regions is indicated by arrows. The proteolytic cleavage site between SU and TM is highlighted in red letters. The C-terminal region of MSRV Env, likely resulting from a recombination event with a HERV-W locus on chromosome 5p12 [see text and 24], is highlighted in yellow. The N-terminal fragment of Syncytin-1 and the C-terminal fragment of Xq22.3 Env used for generation of the anti-Syncytin-1 and anti-Xq22.3 Env polyclonal rabbit antisera are underlined.

We previously found that formerly published MSRV *env *sequences (AF331500, AF127228) are highly similar to a HERV-W *env *element located on the negative strand of human chromosome Xq22.3 (nucleotides 106,182,017-106,184,757, March 2006 human genome assembly) [24]. The Xq22.3 HERV-W locus represents a HERV-W processed pseudogene [8] and consists of 3' portions of the *pol *gene, the complete *env *gene, and U3 and R regions of the 3'LTR (Figure 1A). The primary sequence of the Env protein encoded by Xq22.3 HERV-W *env *is shown in Figure 1B.

To analyze the coding capacity of Xq22.3 HERV-W *env*, we PCR-amplified from human genomic DNA a 1862 bp sequence beginning at the ATG at codon 68 of Xq22.3 HERV-W *env *and containing the putative 475 amino acid HERV-W *env *ORF as well as the 3'LTR portions (Figure 1A). The amplicon was cloned into phCMV, a eukaryotic expression vector under the control of a strong hCMV promotor, generating phCMV-Xq22.3 Env. Another expression plasmid (phCMV-Xq22.3 Env FL) harboring an 2134 bp insert comprising the full-length Xq22.3 HERV-W *env *sequence was created similarly (Figure 1A).

**Figure 2:**
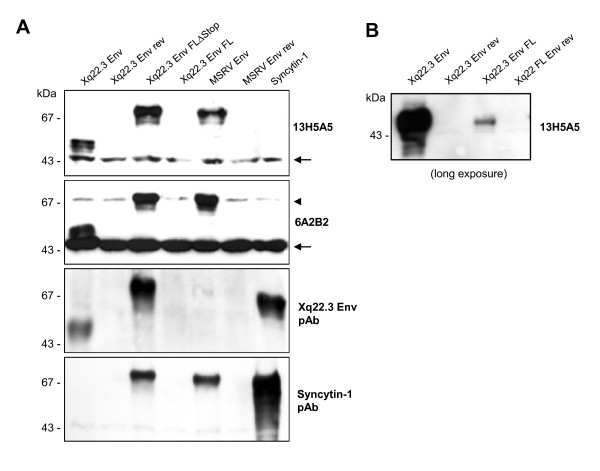
**Eukaryotic expression of Xq22.3 Env**. (A) HeLa cells were transfected with three different Xq22.3 Env constructs as well as MSRV Env, and Syncytin-1. Xq22.3 Env rev and MSRV Env rev contain the respective sequences in reverse orientation and were used as controls. Forty-eight hours post transfection protein expression was studied by Western blot using the indicated primary antibodies. The arrow marks a nonspecific band of about 43 kDa seen in immunoblots incubated with mAbs 13H5A5 and 6A2B2. Another nonspecific band of about 70 kDa observed in immunoblots incubated with 6A2B2 is indicated by an arrowhead. (B) Prolonged exposure of the blot membrane to demonstrate expression of a protein in HeLa cells transfected with Xq22.3 Env FL but not in HeLa cells transfected with a control plasmid containing Xq22.3 Env in reverse orientation (Xq22.3 FL Env rev). The Xq22.3 Env lane, which was included for comparison revealed bands ranging between ~40 to ~53 kDa after overexposure.

HeLa cells were transfected with HERV-W Env plasmids for 48 hours, and protein expression was subsequently analyzed by immunoblots. In whole protein lysates from phCMV-Xq22.3 Env-transfected cells, a mAb (13H5A5) directed against an epitope in the SU domain of MSRV Env [15] detected a protein of ~53 kDa and two smaller proteins of ~50 and ~48 kDa (Figure 2A, top panel, left lane). The molecular weight of the ~53 kDa protein is compatible with the calculated weight (52.9 kDa) of a 475 amino acid HERV-W Env protein with a translational start at the ATG at codon 68. In lysates from phCMV-Xq22.3 Env FL-transfected cells, a ~48 kDa protein became detectable only after prolonged exposure of the blot membranes (Figure 2B). No HERV-W Env proteins were observed in HeLa cells transfected with control plasmids containing inserts in antisense orientation. Weaker expression from phCMV-Xq22.3 Env FL, as compared to phCMV-Xq22.3 Env, may possibly be due to the greater distance between the CMV promotor and the translational start site in this plasmid. In addition to the start codon at position 68, further in-frame ATGs are present at positions 80, 91, 114, and 188 of Xq22.3 HERV-W *env *(Figure 1B), with calculated molecular masses of the resulting proteins of 51.5, 50.2, 47.8, and 39.6 kDa, respectively. Additional smaller proteins observed for phCMV-Xq22.3 Env (Figure 2A and 2B) are thus compatible with Xq22.3 HERV-W Env proteins with a translational start at in-frame ATGs within Xq22.3 HERV-W *env*. In sum, these data demonstrate that Xq22.3 HERV-W *env *has retained a coding capacity for an N-terminally truncated HERV-W Env protein that can be expressed *ex vivo*.

### Reconstitution of full-length Xq22.3 HERV-W Env

We generated an expression plasmid (phCMV-Xq22.3 Env FLΔStop) with an uninterrupted ORF for a full-length 542 amino acid Xq22.3 HERV-W Env protein by reversing the stop codon (TGA) at position 39 of Xq22.3 HERV-W *env *into a tryptophan residue (TGG) (Figure 1A). For comparative analysis with phCMV-Xq22.3 Env FLΔStop we included plasmid phCMV-MSRV Env (pV14), containing the AF331500 MSRV *env *sequence. The structure and possible origin of the AF331500 MSRV *env *sequence were previously discussed in detail [24]. Finally, since Synyctin-1 currently represents the only known functional and thoroughly characterized HERV-W Env protein [29], we also employed the phCMV-Syncytin-1 (PH74) expression vector in this investigation.

Remarkably, reversion of the stop codon in Xq22.3 HERV-W *env *resulted in the expresssion of a ~75 kDa Xq22.3 HERV-W Env protein, as detected by mAb 13H5A5 (Figure 2A, top panel). This antibody also confirmed expression of MSRV Env, with both Xq22.3 Env FLΔStop and MSRV Env proteins having similar molecular weights. Of note, mAb 13H5A5 did not detect Syncytin-1. However, a polyclonal rabbit antibody (pAb) against Syncytin-1 readily recognized Syncytin-1 (Figure 2A, bottom panel). The anti-Syncytin-1 pAb, which is directed against the N-terminus of Syncytin-1, did not cross-react with Xq22.3 Env, further corroborating that Xq22.3 Env is an N-terminally truncated protein. The observed molecular weight of Syncytin-1 is compatible with results from Cheynet *et al*. [29] who reported the full-length Syncytin-1 precursor to be synthesized as a glycosylated 73 kDa protein. It follows that the proteins of approximately similar weight seen for Xq22.3 Env FLΔStop and MSRV Env represent complete HERV-W Env precursor proteins as well. Altogether, reversion of the N-terminal stop codon in Xq22.3 *env *results in the expression of a "resurrected", untruncated, full-length Xq22.3 HERV-W Env precursor protein.

### Specificities of different anti-HERV-W Env antibodies for HERV-W Env constructs

In addition to mAb 13H5A5 and the anti-Syncytin-1 pAb, we also studied the specificity for HERV-W Env proteins of a pAb directed against the 80 C-terminal amino acids of Xq22.3 HERV-W Env. This pAb was generated with the aim of producing a polyclonal rabbit serum that specifically targets Xq22.3 Env. The C-terminal region of Xq22.3 Env was chosen as it displays a number of residues different from MSRV Env and Syncytin-1 (Figure 1B). Indeed, the anti-Xq22.3 Env pAb detected Xq22.3 Env and Xq22.3 Env FLΔStop, but only very weakly MSRV Env (Figure 2A, second panel from bottom). However, it cross-reacted with Syncytin-1, which precluded its use as a tool for exclusive detection of Xq22.3 Env.

We also investigated specificity of mAb 6A2B2 for proteins produced by the different HERV-W Env expression vectors. In our hands, 6A2B2 did detect Xq22.3 Env, Xq22.3 Env FLΔStop, and MSRV Env, but not Syncytin-1 (Figure 2A, second panel from top). A band of ~43 kDa was additionally observed in blots developed with 6A2B2, and infrequently also in blots developed with 13H5A5. This ~43 kDa band was judged unspecific as it was also detected in lysates from a cell line (B95.8) derived from a new world monkey that lacks HERV-W [30] (data not shown).

**Figure 3:**
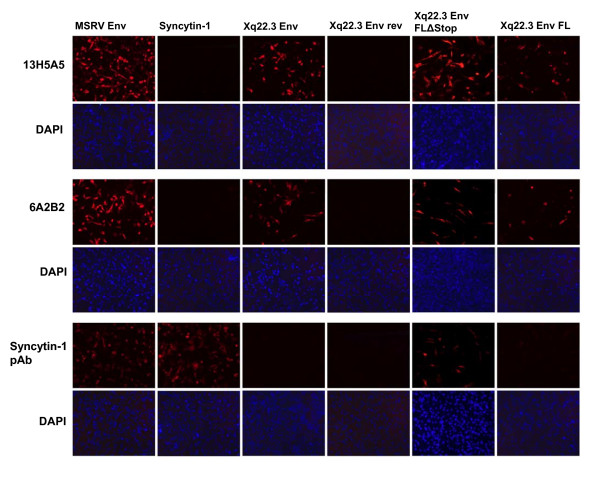
**Immunofluorescence analysis of HeLa cells transiently transfected with expression vectors for HERV-W Env proteins**. HeLa cells were transfected for 24 hours with the indicated expression vectors. Xq22.3 Env rev is a control plasmid which contains the Xq22.3 Env sequence in reverse orientation. Immunocytochemistry was performed on fixed and permeabilized cells with mAbs 13H5A5 and 6A2B2, as well as an anti-Syncytin-1 pAb. Cell nuclei were stained with 4',6'-diamidino-2-phenylindole (DAPI). Magnification × 160.

Expression of the different HERV-W Env proteins and specificity of the different HERV-W Env antibodies for the various HERV-W Env proteins were also investigated by immunocytochemistry. As shown in Figure 3, results obtained by immunocytochemistry were consistent with the immunoblot data. Noteworthy, the protein encoded by phCMV-Xq22.3 Env FL was readily detectable by immunocytochemistry, which likely reflects the higher sensitivity of immunocytochemistry as compared to immunoblots, and further confirms that phCMV-Xq22.3 Env FL has coding capacity.

To summarize the specificities, as established by immunoblot and immunocytochemistry, of all antibodies employed in this work, mAbs 13H5A5 and 6A2B2 detected Xq22.3 Env, Xq22.3 Env FLΔStop, and MSRV Env, but not Syncytin-1. The Xq22.3 Env pAb recognized Xq22.3 Env, Xq22.3 Env FLΔStop, and Syncytin-1, but only very weakly MSRV Env. Finally, the anti-Syncytin-1 pAb reacted with Syncytin-1, Xq22.3 Env FLΔStop, and MSRV Env, but not with Xq22.3 Env.

### Xq22.3 Env is unglycosylated, does not form oligomers, and is not located to the cell surface

**Figure 4:**
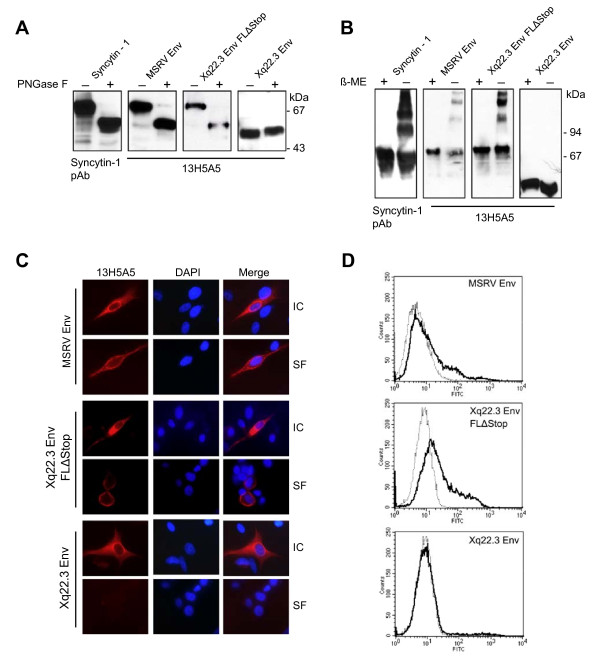
**Comparative characterization of Xq22.3 Env, Syncytin-1, MSRV Env, and reconstituted full-length Xq22.3 Env**. (A) Protein lysates from HeLa cells transfected with the indicated HERV-W Env vectors were treated (+) or not treated (-) with peptide-*N*-glycosidase (PNGase F) to investigate glycosylation of the different HERV-W Env proteins. (B) Protein lysates were generated under reducing (+) or non-reducing (-) conditions to study oligomerization of HERV-W Env proteins. Immunoblots were incubated with the indicated primary antibodies. (C) HeLa cells were grown on microscope slides and transfected with MSRV Env, Xq22.3 Env FLΔStop, and Xq22.3 Env. Surface (SF) expression of the respective proteins was investigated by immunocytochemistry of living, unfixed, and unpermeabilized cells. Intracellular (IC) expression was analyzed in fixed and permeabilized cells. Monoclonal antibody 13H5A5 was used as primary antibody. Magnification × 1000. (D) Flow cytometry was performed on HeLa cells transfected with MSRV Env, Xq22.3 Env FLΔStop, and Xq22.3 Env (black lines) or Xq22.3 Env rev (dotted line) as control. Monoclonal antibody 13H5A5 was used as primary antibody.

Syncytin-1 has been reported to be a moderately glycosylated protein with seven N-linked glycosylation sites [29]. By analogy, we studied the glycosylation pattern of the different HERV-W Env constructs using peptide-*N*-glycosidase (PNGase F) digestion. In agreement with previous findings [29], PNGase F treatment reduced the molecular mass of Syncytin-1 by about 20 kDa (Figure 4A). A similar reduction was observed for Xq22.3 Env FLΔStop and MSRV Env, demonstrating that these proteins are glycosylated in a pattern similar to Syncytin-1. However, PNGase F treatment did not reduce the molecular mass of Xq22.3 Env, indicating that this protein is unglycosylated.

To analyze the capacity of the various HERV-W Env proteins to form oligomers, immunoblots were carried out under reducing and non-reducing conditions. Non-reducing conditions resulted in several high molecular weight bands for Syncytin-1, Xq22.3 Env FLΔStop, and MSRV Env, but no such oligomeric complexes could be observed for Xq22.3 Env (Figure 4B). While the exact composition of those higher molecular weight complexes remains to be determined, our results indicate that Syncytin-1, Xq22.3 Env FLΔStop, and MSRV Env can oligomerize, whereas Xq22.3 Env cannot.

Surface expression of MSRV Env, Xq22.3 Env FLΔStop, and Xq22.3 Env was studied by immunocytochemistry of living, unfixed, and unpermeabilized HeLa cells transfected with respective constructs. Intracellular expression was analyzed in parallel in fixed and permeabilized cells. Whereas MSRV Env and Xq22.3 Env FLΔStop were clearly detectable at the cell surface and in the cytoplasm, Xq22.3 Env was only located in the cytoplasm, suggesting that Xq22.3 Env is not transported to the plasma membrane (Figure 4C). The results obtained by immuncytochemistry were confirmed by flow cytometry experiments in which surface expression was likewise only detectable for MSRV Env and Xq22.3 Env FLΔStop, but not for Xq22.3 Env (Figure 4D).

### A single amino acid mutation inhibits cleavage of HERV-W Env Xq22.3 into SU and TM subunits

**Figure 5:**
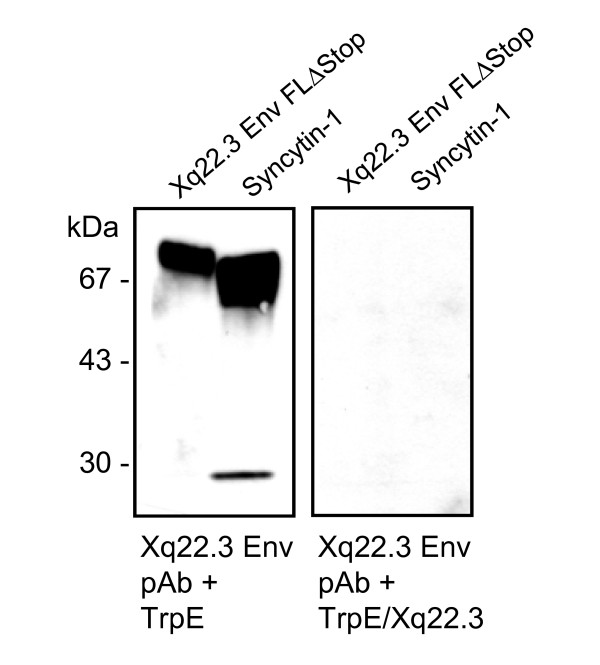
**Reconstituted full-length Xq22.3 Env is not cleaved into SU and TM domains**. Protein lysates of HeLa cells transfected with Xq22.3 Env FLΔStop or Syncytin-1 were analyzed by immunoblot using the anti-Xq22.3 Env pAb as primary antibody, which recognizes the C-terminus of the TM region of Xq22.3 Env and Syncytin-1 (see Figure 2B and text). To confirm the specificity of observed bands, the Xq22.3 pAb was preadsorbed with either TrpE alone or TrpE fused to the C-terminal amino acid fragment of Xq22.3 Env which was used for generation of the anti-Xq22.3 Env pAb.

Cleavage of retroviral Env proteins into SU and TM moieties occurs at a consensus furin cleavage site with the canonical motif R/K-X-R/K-R. While this motif is present in Syncytin-1 (RNKR), a single amino acid of this motif is mutated in Xq22.3 Env and MSRV Env (HNKR) (Figure 1B), suggesting that Xq22.3 Env and MSRV Env might not be properly cleaved. The fact that the anti-Xq22.3 Env pAb, directed against the C-terminal TM region of Xq22.3 Env, cross-detected Syncytin-1 enabled us to use this serum as a tool for studying cleavage of Syncytin-1 and Xq22.3-Env-FLΔStop. In lysates from HeLa cells transfected with Syncytin-1, the anti-Xq22.3 pAb recognized a protein of a little less than 30 kDa, most likely corresponding to the cleaved TM domain of Syncytin-1 (Figure 5, left panel). Conversely, a TM-representing protein was not detected for Xq22.3 Env FLΔStop, indicating that this protein is not cleaved to similar extent as Syncytin-1 into SU and TM subunits. Preadsorption of the anti-Xq22.3 pAb proved the specificity of the observed bands (Figure 5, right panel).

### MSRV Env, Xq22.3 Env FLΔStop, and Xq22.3 Env do not induce syncytia in HeLa cells

**Figure 6:**
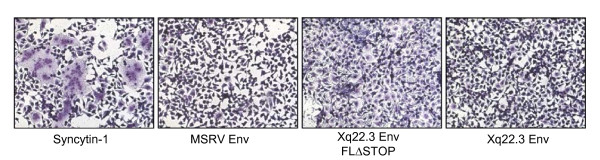
**Syncytin-1 but not MSRV Env, Xq22.3 Env FLΔStop, or Xq22.3 Env induces syncytia in HeLa cells**. HeLa cells were transfected with the indicated HERV-W Env constructs and subsequently stained with May-Grünwald and Giemsa solutions to visualize syncytia formation. Multinucleated giant cells (syncytia) were only detectable in cells transfected with Syncytin-1. Magnification × 250.

Syncytin-1 is a highly fusogenic protein that induces syncytia when expressed in cells that express the type D mammalian retrovirus receptor [11]. We thus analyzed whether MSRV Env, Xq22.3 Env FLΔStop, or Xq22.3 Env may cause formation of syncytia as well. As expected, HeLa cells transfected with Syncytin-1 displayed prominent multinucleated syncytia (Figure 6). In contrast, syncytia were not formed in cells transfected with MSRV Env, Xq22.3 Env FLΔStop, or Xq22.3 Env. This result was somewhat anticipated as the capacity of Syncytin-1 to fuse cells relies on a four amino deletion in the intracytoplasmic TM region of Syncytin-1 [28], and this deletion is neither present in MSRV Env nor in Xq22.3 Env (see also Figure 1B). In addition, proper cleavage into SU and TM domains is required for fusogenicity of Syncytin-1 [29]. Absence of the fusogenic four amino acid deletion and lack of cleavage (Figure 5) therefore sufficiently explain the inability of Xq22.3 HERV-W Env proteins to induce syncytia.

### RNA transcripts from the Xq22.3 HERV-W *env *locus have the correct orientation for translation of a Xq22.3 HERV-W Env protein

**Figure 7:**
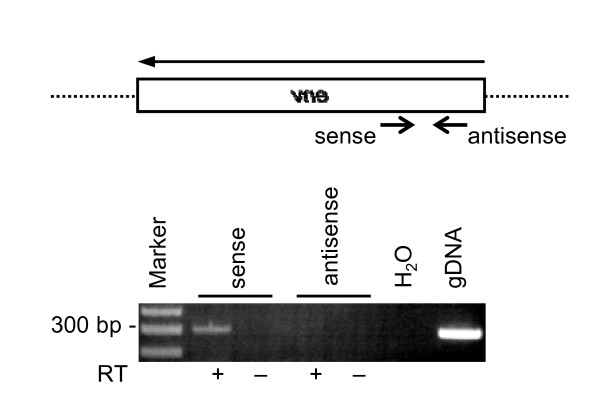
**The Xq22.3 HERV-W *env *locus is transcribed in a sense orientation**. The direction of RNA transcripts from the Xq22.3 HERV-W *env *locus was determined by reverse transcription using strand-specific first strand cDNA synthesis prior to PCR. The localization of the strand-specific primers (depicted by small arrows) relative to the Xq22.3 HERV-W *env *transcript is shown on top. Total RNA isolated from human PBMC was subjected (+) or not (-) to reverse transcription (RT) using either the sense or antisense primer as strand specific primer in the RT reaction. Subsequent amplification by PCR was performed employing both sense and antisense primers. The expected size of the amplified fragment is 305 bp. H_2_O, PCR negative control. Human genomic DNA (gDNA) served as positive control.

The finding that Xq22.3 HERV-W Env can be expressed *ex vivo *opens the possibility that the protein might also be expressed *in vivo*. Transcription of mRNA from the Xq22.3 HERV-W *env *locus is an essential prerequisite for such an expression. By using RT-PCR followed by cloning and sequencing of PCR products, we have previously shown that the Xq22.3 HERV-W *env *locus is indeed transcribed in human PBMC [24]. However, since the reverse transcriptase step in that study involved random hexanucleotide primers it remained to be confirmed that the Xq22.3 HERV-W *env *locus is transcribed in a sense direction, allowing for subsequent translation of Xq22.3 HERV-W Env protein. To clarify this point we performed strand specific reverse transcriptase reactions using primers specific for either sense or antisense transcripts from the Xq22.3 HERV-W *env *locus. Indeed, Xq22.3 HERV-W *env *mRNA is transcribed in a sense orientation with respect to the Xq22.3 HERV-W *env *gene; that is, it has the correct orientation for subsequent translation into a protein (Figure 7). Cloning and sequencing of the respective amplicon (Figure 7, lane 2) confirmed that it originated from Xq22.3 HERV-W *env *(data not shown).

### Monoclonal antibody 6A2B2 detects an antigen expressed in placenta and acute MS lesions

**Figure 8:**
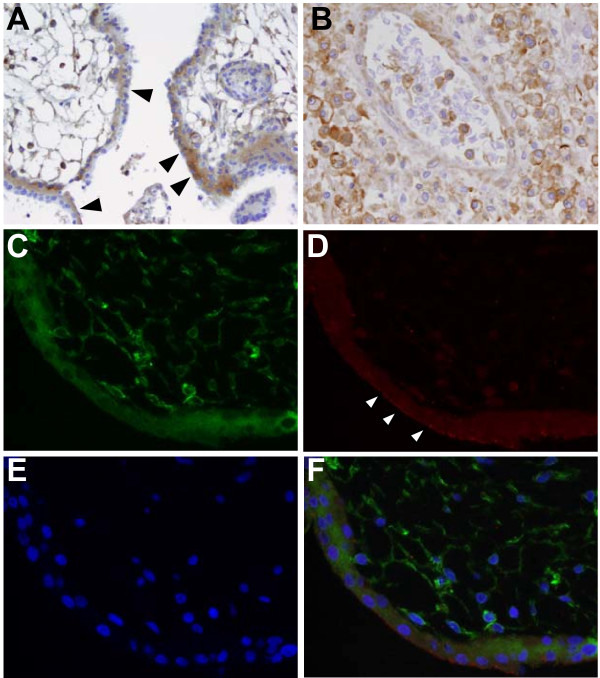
**The monoclonal anti-HERV-W Env antibody 6A2B2 recognizes an antigen expressed in placenta and acute inflammatory MS lesions**. Immunohistochemistry was performed with mAb 6A2B2 on human placenta (A) and an acute MS lesion (B). Arrowheads in A point to syncytiotrophoblast cell layer. Strong staining with 6A2B2 was seen in a case of fulminant MS in activated microglia/macrophages, mononuclear cells, and endothelial cells (B). Magnification × 200 (A), × 400 (B). Double immunofluorescence was carried out on placenta tissue (C-F), with mAb 6A2B2 (C, green) and anti-Syncytin-1 pAb (D, red). Cell nuclei were counterstained with DAPI (E, blue). Merged image (F). White arrowheads in D highlight membrane-associated staining. Magnification × 400 (C-F).

Previous studies have demonstrated that mAb 6A2B2, which has been raised against a 379 amino acid fragment that except for two C-terminal amino acid exchanges is identical to the Xq22.3 HERV-W Env amino acid sequence [24], reacts with an antigen that is expressed in human placenta as well as in inflammatory brain lesions from patients with MS [11,12,16,18]. Having characterized the specificity of mAb 6A2B2 extensively in the present work (Figures 2A, 3), we wanted to reconfirm those findings. Positive immunoreactivity of the syncytiotrophoblast cell layer as well as immunoreactivity of cells within the mesenchyme was observed in human placental tissue stained with mAB 6A2B2 (Figure 8A). Staining with mAb 6A2B2 of an actively demyelinating plaque from a patient with fulminant MS revealed strongly positive immunoreactivity in activated microglia/macrophages, mononuclear cells, and endothelial cells (Figure 8B). To further define the antigen detected by mAb 6A2B2 in placenta, double immunofluorescence was performed with mAb 6A2B2 and anti-Syncytin-1 pAb (Figure 8C-F). Similar to results from conventional immunohistochemistry, mAb 6A2B2 showed a diffuse cytoplasmic staining of the syncytiotrophoblast cell layer as well as of cells within the placental mesenchyme (Figure 8C). In contrast, immunoreactivity of anti-Syncytin-1 pAb was most prominent at the menbrane of the syncytiotrophoblast (Figure 8D). As also indicated by the overlay (Figure 8F), these data suggest that mAb 6A2B2 and the anti-Syncytin-1 pAb recognize different antigens in placental tissue.

## Discussion

We herein show that a processed HERV-W pseudogene which is located on chromosome Xq22.3 and discloses an N-terminally truncated 475 amino acid long *env *ORF can produce an N-terminally truncated HERV-W Env protein *ex vivo*. We named this protein N-Trenv (for N-terminally truncated Env). By analogy to the ERVWE1 gene encoding Syncytin-1, we propose to designate the Xq22.3 HERV-W *env *gene that encodes N-Trenv ERVWE2. While the ERVWE1 gene has been the only HERV-W *env *locus shown to be capable of producing a protein so far, our results establish Xq22.3 HERV-W *env *(ERVWE2) as the second HERV-W *env *element in the human genome that has retained coding capacity.

Viral membrane glycoproteins, such as retroviral Env proteins, are normally synthesized in the endoplasmic reticulum [31]. Targeting of nascent polypeptide chains of retroviral Env proteins to the endoplasmic reticulum membrane is brought about by a short stretch of amino acids at the N-terminus of the protein, the so-called signal peptide [32]. Due to its N-terminal truncation N-Trenv lacks a signal peptide and is therefore very likely synthesized on free ribosomes. Consequently, N-Trenv is not expected to undergo the usual maturation steps of full-length retroviral Env proteins. Indeed, N-Trenv is an unglycosylated protein, that does not form oligomers and is not transported to the cell surface. While functional properties of N-Trenv are currently unknown, those features suggest that in terms of function N-Trenv may behave quite differently from full-length retroviral Env proteins. Remarkably, reversion of the premature stop at codon 39 in ERVWE2 "resurrected" full-length Xq22.3 HERV-W Env protein which then became glycosylated, formed oligomers, and was expressed at the cell surface, just like the full-length HERV-W Env protein Syncytin-1 [29]. A single nucleotide difference in ERVWE2 can therefore dramatically alter properties of the ERVWE2 gene product. Nevertheless, unlike Syncytin-1, reconstituted Xq22.3 HERV-W Env does not appear to be cleaved into SU and TM domains, due to an amino acid mutation in the furin cleavage motif. As cleavage is prerequisite for proper Env function, it is doubtful whether reconstituted Xq22.3 HERV-W Env could represent a fully functional retroviral Env protein that confers infectivity on retrovirus particles, as it was shown for Syncytin-1 [33].

Given the fundamental changes in the properties of N-Trenv resulting from elimination of the premature stop codon in ERVWE2, one may speculate whether a suppression of this stop codon could occur in human beings *in vivo *leading to re-expression of full-length Env with possible functional consequences. Furthermore, it would be interesting to know whether there exist ERVWE2 alleles in the human population that lack the stop codon. An approach to test this hypothesis would be a genetic one, with a screening for mutations in the ERVWE2 stop codon in the general population, or at a more refined level, in certain patient groups, e.g. individuals suffering from MS.

Others and we have previously shown expression of Xq22.3 HERV-W *env *transcripts in human PBMC [24,26,27]. Using strand-specific reverse transcription, we here confirm that Xq22.3 HERV-W *env *is transcribed in the correct orientation for subsequent translation into a protein. The overall structure of Xq22.3 HERV-W *env *transcripts, such as location of upstream transcription initiator(s), downstream polyadenylation signals, and potential splicing of transcripts, must be addressed in separate studies.

An obviously important question is whether N-Trenv may be expressed in the human organism *in vivo*. As our analysis showed, mAb 13H5A5 appears to specifically detect N-Trenv, but not Syncytin-1, and could thus be suited for immunohistochemical studies addressing *in vivo *expression of N-Trenv. Indeed, in a previous immunohistochemical study mAb 13H5A5 was shown to detect an antigen expressed in microglia, lymphocytes, and endothelial cells in formalin-fixed, paraffin-embedded normal human brain tissue and inflammatory MS brain lesions, which were pretreated for antigen retrieval [13]. These data point towards the possibility that N-Trenv could be expressed in normal and diseased human brain *in vivo*. Nevertheless, providing definitive proof that the protein product of a specific HERV locus is expressed *in vivo *is not a simple task. For instance, it cannot be excluded that besides ERVWE2 further defective HERV-W *env *loci in the human genome may have the capacity to express incomplete HERV-W Env proteins that might accidentally contain the epitope recognized by mAb 13H5A5. If this was the case, it would be quite challenging to dissect from which specific HERV-W *env *locus the observed proteins precisely originate. Our present and previously reported findings do therefore not provide formal prove that N-Trenv is expressed *in vivo*, underscoring the complexity of investigations on HERV protein expression *in vivo*.

Since mAb 6A2B2 was raised against a 379 amino acid fragment that is identical to the N-Trenv amino acid sequence except for two C-terminal amino acid exchanges [24], detection of N-Trenv, reconstituted full-length Xq22.3 Env, and MSRV Env by 6A2B2 was expected. However, in our hands 6A2B2 did not recognize Syncytin-1. This was an unanticipated finding as 6A2B2 has previously been reported to detect Syncytin-1 [11,28,29]. A possible explanation for this discrepancy could be that 6A2B2 recognizes an epitope that is conformational or only exhibited by oligomerized forms of Syncytin-1 and that was lost under the experimental conditions applied in our study. Consistent with this idea, in a previous work which reported detection of Syncytin-1 by 6A2B2 in an immunoblot, the respective experiment was carried out under non-reducing conditions [29]. Also consistent with this idea, mAb 6A2B2 was previously shown to react with transiently expressed Syncytin-1 in flow cytometry experiments, where conformational epitopes should be detectable [29].

However, in double immunofluorescence experiments of placental tissue with mAb 6A2B2 and anti-Syncytin-1 pAb both antibodies revealed different staining patterns. Staining with anti-Syncytin-1 pAb was most prominent at the syncytiotrophoblast membrane. Consistent with the detection of Syncytin-1 by the anti-Syncytin-1 pAb in immunoblots and immunocytochemistry, and Syncytin-1 being a membrane-associated protein, this finding is compatible with the detection of Syncytin-1 at the syncytiotrophoblast membrane by the anti-Syncytin-1 pAb. In contrast, membrane-associated immunoreactivity was not observed with mAb 6A2B2 which revealed a more diffuse cytoplasmic staining of the syncytiotrophblast cell layer as well as of cells within the placental mesenchyme. Altogether, these data suggest that, in keeping with our findings on the specificity of mAb 6A2B2 for HERV-W Env proteins in immunoblots and immuncytochemistry, mAb 6A2B2 might also recognize an antigen that is different from Syncytin-1 in immunohistochemistry. It follows that the immunoreactivity observed in MS lesions stained with mAb 6A2B2 in former studies [12,16,18] and this work may indicate expression of an HERV-W Env antigen different from Syncytin-1 in MS brain lesions. Similar to what has been discussed above for mAB 13H5A5, while the precise identity of the antigen recognized by mAb 6A2B2 in placental tissue and MS brain lesions remains to be clarified, N-Trenv is one possibility.

Finally, it is well established that some HERV loci in the human genome have the capacity to produce proteins that can be expressed under physiological but also pathological conditions [7,34]. Research in this field has primarily focused on those few HERV elements that harbor intact ORFs coding for complete retroviral proteins [10]. Nevertheless, there may be instances in which short proteins also produced by highly defective HERV loci may exert a biologically significant function [35]. Our present findings further strengthen the idea that incomplete or truncated HERV loci also may be capable of producing pieces of proteins.

## Conclusions

In summary, we show that a partially defective HERV-W *env *gene on chromosome Xq22.3, that we propose to name ERVWE2, can produce *ex vivo *an N-terminally truncated HERV-W Env protein, named N-Trenv, and has thus retained a coding capacity. Our own and previously published data are compatible with the possibility that N-Trenv could also be expressed *in vivo*, although formal proof is still required. While most of the numerous HERV elements in the human genome are defective and have therefore been considered functionless, our present findings suggest that defective HERV elements in the human genome may be capable of also producing partial HERV proteins. It is tempting to speculate that those HERV protein fragments could play a role in human physiology or pathology.

## Methods

### Plasmids

Nucleotides 106,181,958-106,184,091 from chromosome × (human genome March 2006 assembly), containing the complete Xq22.3 HERV-W *env *sequence, 71 bp of the 3' end of the *pol *gene, as well as 3' LTR portions were PCR-amplified from human genomic DNA. All primer sequences are available from the authors upon request. The PCR product was cloned in either sense or antisense orientation (as control) into the *Bam*HI site of phCMV, generating phCMV-Xq22.3 Env FL (full-length). To create an expression vector containing a complete Xq22.3 HERV-W *env *ORF (phCMV-Xq22.3 Env FLΔStop), the stop codon (TGA) at codon 39 of the Xq22.3 HERV-W *env *sequence present in phCMV-Xq22.3 Env FL was reverted to tryptophan (TGG) by employing a PCR-mediated approach. Expression plasmid phCMV-Xq22.3 Env, starting at the first in-frame ATG (codon 68) downstream from the stop at codon 39 in Xq22.3 HERV-W *env *and containing the 475 amino acid ORF of Xq22.3 HERV-W *env *and the adjacent 3'LTR sequence was generated by PCR-amplification of nucleotides 106,181,958-106,183,819 of chromosome × from human genomic DNA. The amplicon was introduced in either sense or antisense orientation into the *Bam*HI site of phCMV. Sequencing of phCMV-Xq22.3 Env and phCMV-Xq22.3 Env FL (GATC Biotech, Konstanz, Germany) confirmed identity with the Xq22.3 human genome sequence (March 2006 assembly) and reversion of the stop codon in phCMV-Xq22.3 Env FLΔStop (data not shown). Plasmids phCMV-Syncytin-1 (clone PH74, AF 072506) [11], and phCMV-MSRV Env (clone pV14, AF 331500) [4] were kindly provided by Hervé Perron (Geneuro, Switzerland). As an additional control, we also inserted the MSRV *env *sequence in antisense orientation into phCMV.

### Antibodies

Polyclonal anti-Syncytin-1 rabbit antisera (anti-Syncytin-1 pAb K342 and K343) were generated as previously described [36,37] by immunization of rabbits with a fusion protein consisting of an approximately 6 kDa fragment of the N-terminus of Syncytin-1 and *E. coli *anthranilate synthetase (TrpE). A polyclonal anti-Xq22.3 HERV-W Env antiserum (anti-Xq22.3 Env pAb) was produced similarly by immunizing rabbits with a fusion protein consisting of the 80 C-terminal amino acids (463-542) of Xq22.3 HERV-W Env fused to TrpE (see also Figure 1A). In competition experiments, the anti-Xq22.3 HERV-W Env antiserum was preadsorbed overnight with either bacterially expressed TrpE-Xq22.3 HERV-W Env fusion protein or TrpE alone. The mouse mAb 13H5A5 (IgG1κ), directed against an epitope in the SU domain of MSRV Env [15], and peroxidase labeled mouse mAb 6A2B2 (IgG1κ) were a kind gift from Hervé Perron. As previously discussed in detail, mAb 6A2B2 was raised against a 379 amino acid sequence encoded by MSRV *env *clone AF127228, which is identical to the Xq22.3 HERV-W Env amino acid sequence but for two C-terminal amino acid exchanges [11,24].

### Transient expression and characterization of HERV-W Env proteins

HeLa human cervical carcinoma cells were cultured in Dulbecco's modified Eagle's medium containing 10% FCS (PAA), penicillin (100 U/ml), and streptomycin (100 μg/ml). Cells were maintained at 37° C in a 5% CO_2 _atmosphere. Cells were free of mycoplasma contamination, as confirmed by PCR (Venor GeM, Minerva Biolabs). For transient expression of HERV-W Env proteins, HeLa cells were plated into 6-well plates (5 × 10^5 ^cells/well) and transfected the following day with different HERV-W Env expression plasmids using Lipofectamine 2000 (Invitrogen) according to the manufacturer's instructions. After approximately 48 hours, cells were lysed in sample buffer (125 mM Tris-HCl [pH 6.8], 6% [w/v] sodium dodecyl sulfate [SDS], 10% [v/v] mercaptoproprandiol, 10% [v/v] glycerol), sonicated, and boiled for 5 minutes. To assay for HERV-W Env oligomers, mercaptoproprandiol was omitted from the sample buffer and lysates were not boiled. For immunoblot analyses, 15 μg of protein were separated by SDS-PAGE and transferred onto polyvinylidene difluoride membranes (Millipore). Membranes were probed with primary antibodies as indicated and developed with secondary peroxidase labeled IgG antibodies (Sigma) and enhanced chemoluminescence. To analyze glycosylation of HERV-W Env proteins, 30 μg of protein were denatured at 100°C for 10 minutes and digested with 500 Units PNGase F (New England BioLabs) for 4 hours at 37°C according to the manufacturer's instructions.

### Flow cytometry

HeLa cells transfected with the different HERV-W Env expression vectors were washed with PBS and detached with 0.02% Versene in PBS. Cells were incubated with primary antibody 13H5A5, diluted in FACS buffer (PBS, 2% [v/v] FCS), for 1 hour at 4°C, washed with FACS buffer, and incubated with a fluorescein isothiocyanate-conjugated anti-Mouse IgG secondary antibody (Sigma). Cells were washed, resuspended in isotonic NaCl 1% (v/v) paraformaldehyde (PFA) [pH 7,4], and 10^4 ^cells/sample were analyzed using a Beckton Dickinson FACScan.

### Immunocytochemistry

For immunocytochemistry, HeLa cells were plated at 3 × 10^4 ^cells per well (growth area 0.9 cm^2 ^per well) on microscope slides using flexible 8-well tissue culture chambers attached onto the slides (flexiPerm, Greiner Bio-one). The following day, cells were transfected using Lipofectamine and medium was replaced 4 hours after transfection. For whole cell staining, cells were washed one day later with PBS, fixed with 4% (v/v) PFA in PBS, permeabilized with 0.2% (v/v) Triton-X 100 in PBS, blocked with 10% (v/v) FCS in PBS, and stained with antibodies diluted in PBS/10% FCS. Following incubation with secondary antibody (goat-anti-rabbit IgG Alexa Fluor 568, 1:1000, Molecular Probes), cells were dehydrated in 70%, 80%, and 100% ethanol and mounted with Vectashield (Vector) medium containing 4',6'-diamidino-2-phenylindole (DAPI). For surface staining, living, unfixed, and unpermeabilized cells were stained on ice with primary antibodies as indicated. After incubation with secondary antibody, cells were fixed with PFA. Slides were inspected with a Leitz Aristoplan fluorescence microscope and images were acquired using AxioVision 3.0 software. Digital acquisition parameters and further processing (Corel Photo-Paint 12) were identical for all images.

### Formation of syncytia

HeLa cells were plated at 5 × 10^5 ^cells/per well in 6-well plates and transfected (Lipofectamine 2000) with different HERV-W Env constructs. After 24 hours cells were stained with May-Grünwald and Giemsa solutions to visualize syncytia formation and inspected with a Leitz Aristoplan microscope.

### Immunohistochemistry

Immunohistochemistry was carried out as previously described [38] on formalin-fixed paraffin-embedded post-mortem brain tissue sections from a 76-year old woman with fulminant MS (Marburg's variant) as well as on formalin-fixed paraffin-embedded tissue sections from normal human placenta. For double immunofluorescence Cy2-conjugated anti-mouse-IgG and Cy3-conjugated anti-rabbit IgG (Dianova, Hamburg, Germany) were used as secondary antibodies.

### Strand specific RT-PCR

Total RNA was prepared from PBMC using the RNeasy Mini kit (Qiagen) and eluted into 60 μl of RNase-free H_2_O_dd_. RNA concentration and purity was assessed spectrophotometrically. Contaminating DNA was removed using the TURBO DNA-free Kit (Ambion Inc.) following the protocol for rigorous DNAse treatment as described before [24]. Reverse transcription was carried out using the Xq22.3 HERV-W *env *strand-specific primers Sense 5'-GCTGCTGTACAACCAGTAGCTC-3' and Antisense 5'-TTCTCTTGCCTGACCTTGAAT-3' and SuperScript III reagents (Invitrogen) at 55°C for increased specificity. Negative controls were generated in parallel for each sample by omitting reverse transcriptase from the reaction. PCR was carried out as reported [24] using above mentioned primers. For further verification PCR products were cloned and sequenced as described before [24].

## Competing interests

The authors declare that they have no competing interests.

## Authors' contributions

KR, JM, and NML conceived of the study, participated in its design, and provided funding. CC, SW, GL, and KR carried out the experimental work. CC, SW, GL, JM, and KR analyzed the data. CS carried out and analyzed the immunohistochemical studies. KR drafted the manuscript. All authors read and approved the final manuscript.
